# Modified Nigella Sativa Seeds as a Novel Efficient Natural Adsorbent for Removal of Methylene Blue Dye

**DOI:** 10.3390/molecules23081950

**Published:** 2018-08-05

**Authors:** Souad Rakass, Ahmed Mohmoud, Hicham Oudghiri Hassani, Mostafa Abboudi, Fethi Kooli, Fahd Al Wadaani

**Affiliations:** 1 Chemistry Department, College of Science, Taibah University, Al-Madinah 30002, Saudi Arabia; caadil77@yahoo.co.uk (A.M.); oudghiri_hassani_hicham@yahoo.com (H.O.H.); abboudi14@hotmail.com (M.A.); fwadaani@taibahu.edu.sa (F.A.W.); 2 Département de Chimie, Faculté des Sciences Dhar El Mahraz, Université Sidi Mohamed Ben Abdellah, B. P. 1796 (Atlas), Fès 30003, Morocco; 3 Community College, Taibah University-Al-Mahd Branch, Al-Mahd 42112, Saudi Arabia; fethi_kooli@yahoo.com

**Keywords:** modified nigella sativa seeds, removal, recycling, methylene blue

## Abstract

The aim of this work was to investigate the use of modified nigella sativa seeds (MNS) for removing of methylene blue (MB) dye from aqueous solution. The nigella sativa (NS) seeds have been pre-treated at different temperatures and periods of time. The maximum adsorption of MB was achieved using NS sample washed with distilled water pre-heated at 65 °C for one hour, then ground to 250 µm particle size (MNS-4). Different parameters were modified to optimize the removal process of MB using MNS-4, such as contact times, temperatures, initial dye concentrations, adsorbent doses, and pH of the solution. MNS-4 exhibited a removal efficiency of 99% for initial dye concentrations greater than 800 ppm at pH value of 11. The kinetic study indicated that the removal process follows the pseudo second order model. The removal was spontaneous, endothermic and favorable, and this was indicated by the thermodynamic study. Maximum removal capacity was 194 mg/g as deduced from Langmuir model. The removal efficiency was maintained after four recycle uses. The modified nigella sativa seeds were characterized before, and after adsorption and regeneration by Fourier Transform infrared (FTIR) and scanning electron microscopy (SEM). The data suggested that nigella sativa seeds could be a prospective agent for removing MB from wastewater.

## 1. Introduction

The waste leftover by the textile industry creates a number of problems in the environment owing to the unwanted toxic materials present such as dissolved solids, bases, acids and dyes [1,2]. Dyes are aromatic molecular structures, which are anticipated to be firm & steady and, as a result, are difficult to degrade (making them more recalcitrant to biodegradation) [3,4]. Synthetic dyes have been widely applied to many technological areas, which include the leather tanning, textile industry, food technology, in photo electrochemical cells, paper production, and for hair coloring products [4]. However, because of the major production of synthetic dyes and their broad applications [5], they are highly toxic, harmful and can cause substantial environmental toxic waste and pose a serious hazard to the public health [6,7]. Consequently, scientists have developed various methods of chemical and physical processes for removing different dyes. For example, removal of dyes from waste effluents by ozone oxidation, coagulation by a chemical agent, electrochemical method, and hypochlorite oxidation and adsorption [7,8,9,10]. Adsorption was reported to be an efficacious process for removing dyes from wastewater, and an alternative method compared to other expensive treatment techniques [9,11,12]. It is performed using synthetic [13,14,15,16,17,18] or natural adsorbents [19,20,21,22,23,24]. However, synthetic adsorbents can be costly, as the higher the quality, the greater the expenditure. 

To the present day, several research investigations in the literature have reported removing textile dyes using natural adsorbents, as they are economically attractive and advantageous mainly due to their cheapness and abundant availability [25,26,27,28]. However, there is still a need to find efficient natural adsorbents with high adsorption capacity that can be easily separated and presenting a criteria and performance for recycling and regeneration.

Several researchers were interested to study the adsorption of methylene blue (MB) dyes using cultivated solid wastes such as rice husks, peanut hulls, banana peels, castor seed shells, and gulmohar plants, with dye adsorption capacities of 41, 68, 21, 158, and 186 ppm respectively [28,29,30,31,32,33]. One of the most widely used adsorbents for wastewater management is activated carbon due to its high removal capacity, where it can reach 486 mg/g for the activated carbons, which are derived from agricultural and industrial wastes, and 980 mg/g for commercial activated carbon and coal [34]. However, its use is limited by regeneration issues, the high-cost production, phase separation difficulty and poor mechanical properties [35,36].

Few studies have been reported using nigella sativa (NS) seeds on the removal of dyes [37]. In our case, the NS have been modified physically by heating at different temperatures and periods of time. The main target of this study was to propose an alternative and an efficient natural adsorbent with high adsorption capacity and good recycling and regeneration properties. The nigella sativa seeds are cheap material and easily available in local areas. The MB was used as a model dye due to its wide industrial applications such as a coloring agent for food, cotton, leather, wool and silk [38]. Different effects such as contact time, initial dye concentration, adsorbent dosage, and solution pH on the removal of methylene by modified nigella sativa seeds were investigated. The regeneration using a simple mixture of ethanol and water of modified nigella sativa seeds was also studied.

## 2. Experimental

### 2.1. Materials

Fresh NS seeds were obtained in its natural state from a local supermarket. The MB dye (molecular formula: C_16_H_18_ClN_3_S.xH_2_O, M: 319.85, λ_max_ = 665 nm) was supplied by Panreac, Barcelona, Spain. All the reagents used without purification were high purity analytical grades.

### 2.2. Nigella Sativa Treatment

NS seeds have been modified at different conditions to find out the best conditions for removal of Methylene blue dye, and the samples are assigned as following:

The sample in its natural state (NS), then ground and sieved using 250 µm particle size siever (NS-1)

NS sample ground to 250 µm particle size and heated for one hour at 100 °C (MNS-1).

NS sample ground to 250 µm particle size and heated for twenty-four hours at 100 °C (MNS-2).

NS sample washed with distilled water, then dried and heated for one hour at 65 °C (MNS-3).

NS sample washed with distilled water, dried and heated for one hour at 65 °C and then ground to 250 µm particle size (MNS-4).

### 2.3. Removal Studies

The removal study of MB was carried out in a batch equilibrium of fixed amount of sample (1 g) and volume of known concentrations (100 mL). The mixtures were kept under isothermal conditions at fixed temperature without changing the pH. The solution after equilibrium was collected by filtration using 0.45 µm syringe filters (Whatman), then analyzed by UV-Vis spectrophotometer (Thermo Fisher Scientific, Madison, WI, USA).

The influence of different parameters namely adsorbent doses, contact time, pH and initial dye concentration (Ci) were studied by altering one parameter and keeping the others unchanged. The adjustment of pH was carried with diluted HCl or NaOH (0.1 mol/L) and measured using SCT-BEN-PH-1 pH Meter (JJS, Dallas, TX, USA). 

The equilibrium MB concentration (C_e_ in ppm), was calculated using the standard calibration curve equation of a straight line, A = 0.1865C_e_ + 0.0778.

The following equation is used to calculate the removal percentage of the MB dye:

(1) $$\mathrm{Removal}\;\mathrm{percentage}\;\left(\%\right)=\frac{C_{i}-C_{e}}{C_{i}}\;\times \;100\;$$

The removed amount of MB at equilibrium, (q_e_, mg·g^−1^), is estimated by:

(2) $$q_{e}=\frac{\left(C_{i}-C_{e}\right)}{W}\times V\;$$

where C_e_ is the equilibrium dye concentration (ppm) and C_i_ is the initial dye concentration of (ppm); W is the mass of the adsorbent (g) and V is the volume of the solution (L).

### 2.4. Regeneration Method

The regeneration tests were investigated within the same settings. The spent MNS-4 with an appropriate amount of MB was treated in a mixture of 50 mL:50 mL ethanol and distilled water, at room temperature, while stirring for a period of 60 min. The solid was collected and washed twice with 100 mL distilled water, then dried at 65 °C before the next test.

### 2.5. Characterization

The sample before and after removal, and recycling were analyzed by Fourier transform infrared spectroscopy (FTIR) using IRAffinity-1S Shimadzu spectrometer (Shimadzu, Tokyo, Japan). In the 400–4000 cm^−1^ range, using KBr technique. To examine the texture, shape and the size of the particles, SEM micrographs were obtained using scanning electron microscope (SEM) model Quanta Feg 250 (Thermo Fisher Scientific, Hillsboro, OR, USA). The samples were loaded on a carbon tape. The solution concentration at equilibrium was estimated by the Thermo Scientific Genesys 10S UV-Vis Spectrophotometer (Thermo Fisher Scientific, Madison, WI, USA) at λ_max_ 665 nm.

## 3. Results and Discussion

### 3.1. Removal of MB Dye onto NS Adsorbent

#### 3.1.1. Effect of NS Treatment on Removal of MB dye

**Figure 1:**
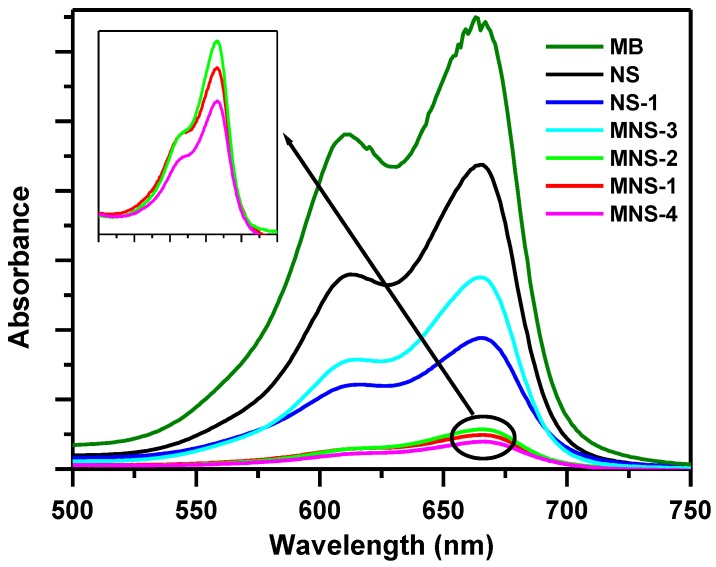
UV spectra of methylene blue (MB) solutions (20 ppm) before and after contact with nigella sativa (NS) and its modified derivatives.

Figure 1 presents the evolution of UV spectra of MB solution (C_i_ = 20 ppm) after contact with nigella sativa (NS) raw material and modified derivatives at different conditions. 

The data indicated the solutions at equilibrium after contact with MNS-1, 2, and 4 samples have the low absorbance in intensity, and indicated that these samples exhibited similar removal capacities (taking in account the experimental errors). This fact indicated that the thermal treatment made easy the accessibility of the active sites to the MB removal process. MNS-4 was selected in further studies.

#### 3.1.2. Effect of Initial Dye Concentration and Contact Time without pH Adjustment

**Figure 2:**
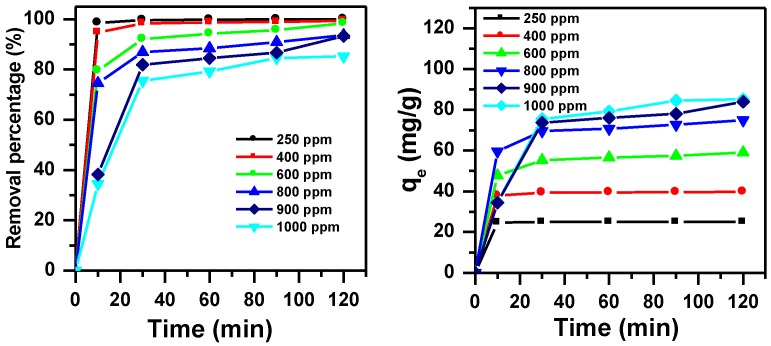
Effect of initial dye concentration and contact time on the removal efficiency of MB using MNS-4.

The effect of the initial MB concentration with contact time on the removal was tested without pH adjustment and presented in Figure 2. The removal of MB dye was rapid in the first 10 min and became gradually constant after 30 min until 120 min. The percentage removal varied from 100% to 85% and the removal capacity was from 25 mg/g to 80 mg/g with different concentrations of 250 ppm to 1000 ppm respectively. The amount of MB removed in the first 10 min could be related to the availability of vacant sites at early stages; however, this number was reduced because of the increase of MB molecules in the vacant sites, leading to a decrease of the removal amount [39].

#### 3.1.3. Effect of pH

**Figure 3:**
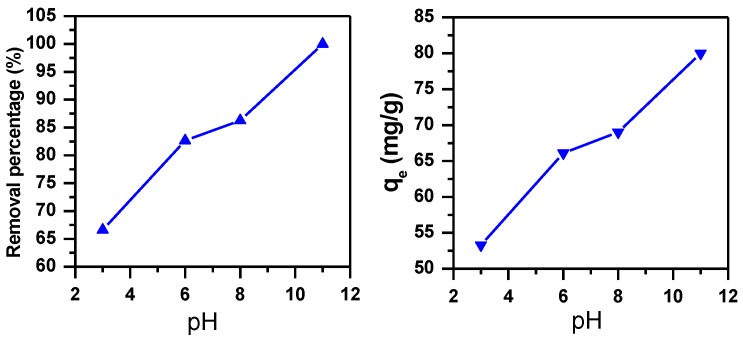
Effect of pH on the removal efficiency of MB using MNS-4 (C_i_ = 800 ppm, m adsorbent = 1 g, contact time = 30 min).

The pH plays an important factor that affects the removal of dyes [40]. Figure 3 shows the removal of MB dye using MNS-4 adsorbent at different pH from 3 to 11. The percentage removal and the removed amount of MB dye improved as pH of the solution was increased. The removal percentage was enhanced from 67% to 100% and the removed capacity increased from 53 mg/g to 80 mg/g, respectively. The high removal of MB at pH values of 6–11 could be ascribed to electrostatic attraction between the negative charge of the sample surface and the positive charge of MB cations, due to the deprotonation of the surface functional groups at higher pH values [39]. The following tests will be carried at pH of = 11.

#### 3.1.4. Effect of Initial Dye Concentration and Contact Time with pH Adjustment

**Figure 4:**
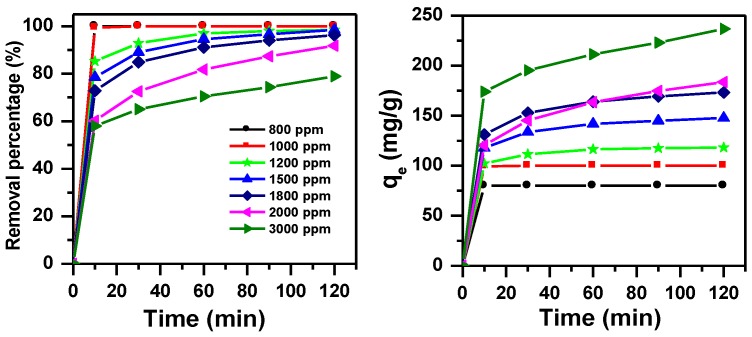
Effect of initial dye concentration on removal efficiency of MB using MNS-4 (m adsorbent = 1 g, pH = 11).

The effect of initial dye concentration with contact time on the removal of MB has been studied again at pH = 11 and for MNS-4 sample, as shown in Figure 4. A maximum removal of 100% was achieved whiten 10 min using C_i_ of 800 and 1000 ppm. For C_i_ values of 1200, and 1500 ppm, the maximum removal percentage (99%) was achieved after 60 min of contact time. For higher C_i_ values than 1800 ppm the maximum value of removal was obtained after 120 min of contact time. In all the cases, the time needed to achieve the maximum removal percentage was shorter compared to solutions without pH adjustment of MB dye.

#### 3.1.5. Effect of Adsorbent Dose

**Figure 5:**
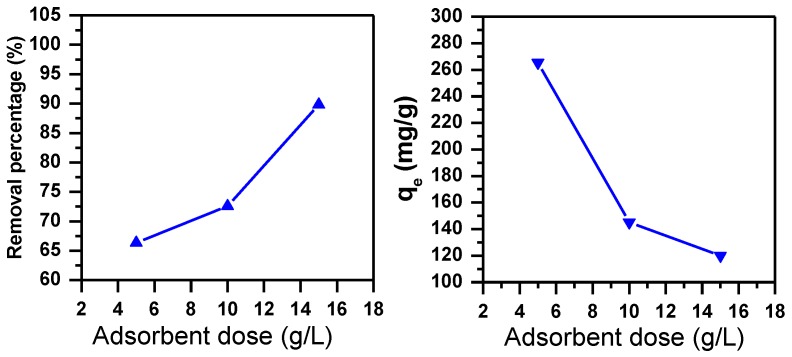
Effect of adsorbent dose on the removal efficiency of MB using MNS-4 (C_i_ = 2000 ppm, contact time = 30 min, pH = 11).

The effect of adsorbent dose on the removal efficiency of MB on MNS-4 was investigated. The removed amount of MB increases with an increasing of adsorbent dose as shown in Figure 5. By increasing the adsorbent dose, the active sites of the adsorbents’ surface area also increased, hence the amount of MB removed increases [41].

#### 3.1.6. Effect of Temperature and Thermodynamic Parameters

**Figure 6:**
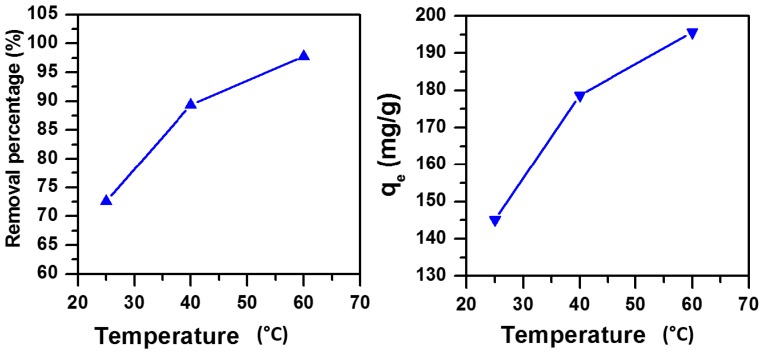
Effect of temperature on the removal efficiency of MB using MNS-4 (C_i_ = 2000 ppm, m adsorbent = 1 g, contact time = 30 min, pH = 11).

Since temperature has a significant effect on the removal of dyes [40], the temperature value was varied from 25 to 60 °C during the removal of MB dye as shown in Figure 6. The removal percentage of MB (C_i_ = 2000 ppm) increased from 73% to 98%, while the removal capacity increased from 145 mg/g to 196 mg/g. In fact, as the temperature increases the activity of the removal adsorbent sites improved with increasing the mobility of the dye molecule [40,42].

**Figure 7:**
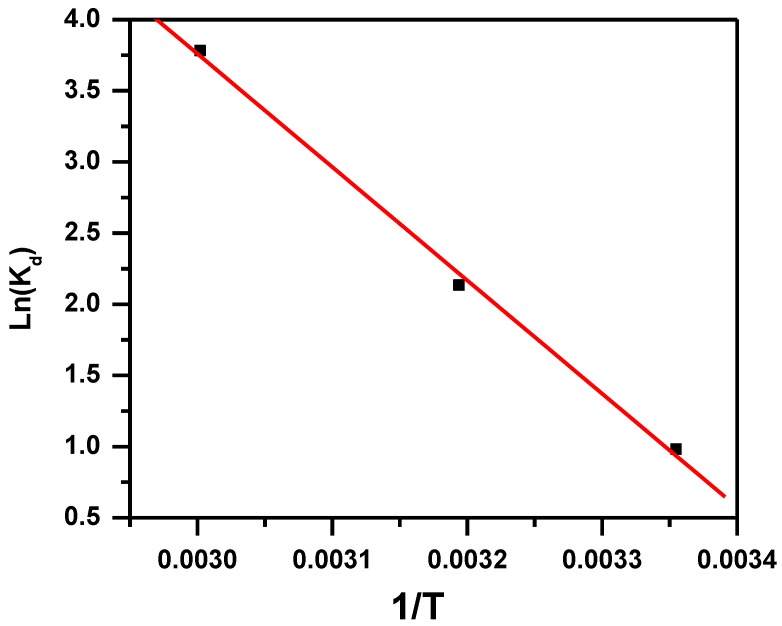
van ’t Hoff plot for the effect of temperature on removal of MB using MNS-4.

Thermodynamic factors are important in the process of adsorption methods [43,44]. The mechanism and the probability of adsorption can be predicted in respect to thermodynamic factors [43]. To determine the thermodynamic parameters, the following equations were used:

(3) $$\Delta G^{o}=-{\mathrm{RTLnK}}_{d}\;$$

(4) $$K_{d}=\frac{C_{a}}{C_{e}}\;$$

(5) $${\mathrm{LnK}}_{d}=\frac{\Delta S^{o}}{R}-\frac{\Delta H^{o}}{\mathrm{RT}}\;$$

where ΔG° is the free energy, R is the gas constant (J·mol^−1^·K^−1^), T is absolute temperature (K), K_d_ is the distribution constant, C_e_ is the equilibrium concentration, C_a_ is the amount of dye adsorbed on the adsorbent of the solution at equilibrium (mol/L), ΔS° is the standard entropy and ΔH° is the standard enthalpy. ∆H° and ∆S° values were obtained from the slope and intercept of plot ln K_d_ against 1/T (Figure 7). ∆G° values were obtained from Equation (3) and presented in Table 1. The adsorption is favorable and spontaneous, indicated by the negative value of ∆G°. The ∆H° value indicates that the removal of MB occurred in a physisorption process as indicated by the positive value of ∆H° (66 KJ mole^−1^) [45]. The increased disorder and randomness at the solid solution interface of MB and MNS-4 is indicated by the positive values of ∆S°. The adsorbate molecules displace the adsorbed water molecules, which results in gaining more translational energy than is lost by the adsorbate molecules, and hence allows occurrence of randomness in the system [31].

### 3.2. Kinetics of Adsorption

The practicality of the process is crucial and so the kinetic study of the adsorption was carried out as it gives evidence about the adsorption mechanism [46].

The kinetics data of the removal of MB by MNS-4 adsorbent was gathered by using intraparticle diffusion kinetic, pseudo-first order and pseudo-second order models.

#### 3.2.1. Pseudo-First-Order Kinetic Model

Lagergren reported Pseudo-first order; Equation (6) describes this model:

(6) $$\mathrm{Ln}\left(q_{e}-q_{t}\right)={\mathrm{Lnq}}_{e}+K_{1}t\;$$

where q_t_ and q_e_ are the removal capacity at time t and at equilibrium, respectively (mg/g), K_1_ represents the rate constant of pseudo-first-order adsorption (1/min).

#### 3.2.2. Pseudo-Second-Order Kinetic Model

Equation (7) describes the pseudo-second order model [47]:

(7) $$\frac{t}{q_{t}}=\frac{1}{K_{2}q_{e}^{2}}+\frac{t}{q_{e}}\;$$

the slope and intercept of the plot t/q_t_ versus t is used for the calculation of this model. Where q_t_ is the amount of MB adsorbed at time t (min) and K_2_ (g·mg^−1^·min^−1^) is the pseudo-second order rate constant.

#### 3.2.3. Intraparticle Diffusion Process

Equation (8) shows the intraparticle diffusion [48]:

(8) $$q_{t}=K_{I}t^{1/2}+I\;$$

where K_I_ (mg/(g·min^0.5^)) and I (mg/g) are the intraparticle diffusion constants, t is the contact time (min), and q_t_ is the removal capacity (mg/g) at time t.

**Figure 8:**
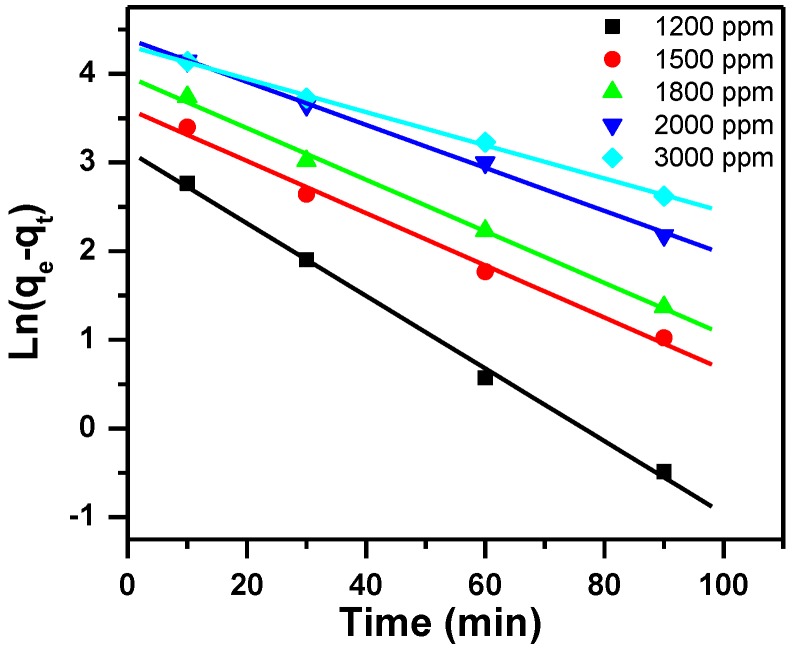
Pseudo-first order plot for the effect of initial dye concentration and contact time on removal of MB using MNS-4.

**Figure 9:**
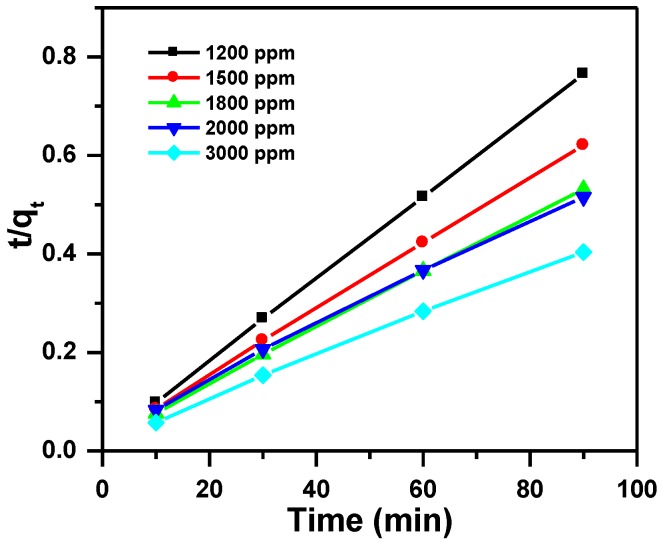
Pseudo-second order plot for the effect of initial dye concentration and contact time on removal of MB using MNS-4.

**Figure 10:**
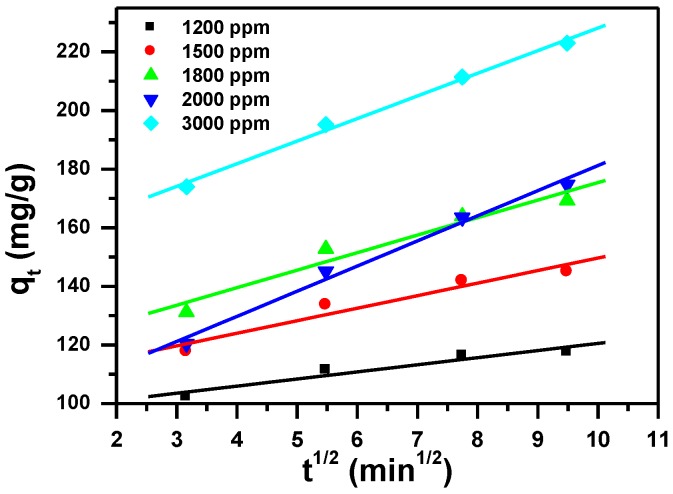
Intra-particle diffusion plot for the effect of initial dye concentration and contact time on removal of MB using MNS-4.

The parameters of the three models are summarized in Table 2, and presented in Figure 8, Figure 9 and Figure 10. The regression correlation coefficients (R^2^) for the three models are between 0.993 and 0.998, between 0.998 and 1.000 and between 0.889 and 0.994, for the different used initial concentrations respectively. Data from the experimental work shows that pseudo-second order model fits well, due to the corresponding R^2^ value close to 1. Furthermore, the experimental values agree with the calculated q_e_ values.

### 3.3. Adsorption Isotherm Models

Adsorption isotherms are important due to their accurate descriptions when designing adsorption processes. Four adsorption models have been tested, such as Freundlich, Langmuir, Temkin isotherm, and Dubinin-Radushkevich models.

#### 3.3.1. Langmuir Isotherm

Langmuir isotherm signifies the equilibrium distribution of the metal ions between the liquid and the solid phases [49]. It defines quantitatively the foundation of a monolayer adsorbate on the outer surface of the adsorbent (on a fixed number of distinctive sites), and once it is complete, no more adsorption takes place. All the sites are vigorously alike, where each site can only hold one ion, and there is no interaction between the ions [50,51]. The maximum removal capacity (q_m_) of the adsorbent is determined by the isotherm data analysis [52]. Equation (9) shows the Langmuir isotherm form [53]:

(9) $$\frac{C_{e}}{q_{e}}=\;\frac{1}{q_{m}K_{L}}+\frac{C_{e}}{q_{m}}\;$$

where C_e_ is the concentration of the MB dye at equilibrium (ppm); q_e_ is the amount of the MB dye adsorbed on the MNS-4 adsorbent at equilibrium (mg/g); K_L_ is the Langmuir constant of adsorption (L/mg); and q_m_ is the maximum removal amount of MB dye onto MNS-4 adsorbent (mg/g).

The following equation explains the equilibrium parameter or dimensionless constant separation factor, R_L_, [54].

(10) $$\;R_{L}=\frac{1}{1+K_{L}C_{i}}\;$$

where K_L_ is the Langmuir constant, and C_i_ is the initial MB concentration, R_L_ values indicate that the removal could be irreversible (R_L_ = 0), favorable (0 < R_L_< 1), linear (R_L_ = 1) or unfavorable (R_L_ > 1) [55].

#### 3.3.2. Freundlich Isotherm

The Freundlich isotherm model can be applied to surfaces supporting sites of varied affinities, or heterogeneous surfaces assuming that stronger binding sites are occupied first and then binding strength decreases with increasing degree of site occupation [56]. Equation (11) shows the Freundlich isotherm form:

(11) $$\;q_{e}=q_{F}C_{e}^{1/n}\;$$

This can be expressed as:

(12) $$\;{\mathrm{Lnq}}_{e}={\mathrm{Lnq}}_{F}+\frac{1}{n}{\mathrm{LnC}}_{e}\;$$

where n (g/L) is the heterogeneity factor and q_F_ (mg^(1−1/n)^L^1/n^g^−1^) is the Freundlich constant. The adsorption capacity and the q_F_ values are related; while the adsorption intensity is related to 1/n value.

#### 3.3.3. The Dubinin-Radushkevich (D-R) Isotherm

Adsorption on heterogeneous and homogeneous surfaces can be described by the D-R isotherm model at low concentration [57].

The following equation shows the linear form of the isotherm.

(13) $$\;{\mathrm{Lnq}}_{e}={\mathrm{Lnq}}_{m}-{K\varepsilon }^{2}\;$$

where ε is the Polanyi potential determined in Equation (14), and K is constant for the sorption energy (mol^2^/kJ^2^):

(14) $$\varepsilon \;=\mathrm{RTLn}\left(1+\frac{1}{C_{e}}\right)\;$$

where R is the Universal gas constant (8.314 J·mol^−1^ K^−1^), T (K) is the temperature and C_e_ (ppm) is the equilibrium concentration of the MB dye left in the solution, and q_m_ is the theoretical saturation capacity.

The following equation represents the mean energy of sorption, E (kJ/mol):

(15) $$\;E=\;\frac{1}{\sqrt{2K}}\;$$

The mechanism of the adsorption can be estimated by using the magnitude of E. Physical forces could affect the adsorption in such cases of E = 8 kJ/mol. Adsorption can be ruled by ion exchange mechanism if E is in the range of 8–16 kJ /mol, whereas adsorption may be subject to particle diffusion for the value of E = 16 kJ/mol, [44,58].

#### 3.3.4. Temkin Model

This isotherm contains a factor that explicitly taking into the account of adsorbent-adsorbate interactions. It further implies that the heat of adsorption of all the molecules in the layer would decrease linearly with the coverage involved in this interaction [49]. The following equation is used for the Temkin isotherm.

(16) $$\;q_{e}=B_{T}{\mathrm{LnA}}_{T}+B_{T}{\mathrm{LnC}}_{e}\;$$

where B_T_ = R_T_/b_T_, b_T_ is the Temkin constant related to heat of sorption (J/mol), A_T_ is the Temkin isotherm constant (L/g), R is the gas constant (8.314 J/mol K), and T is the absolute temperature (K).

**Figure 11:**
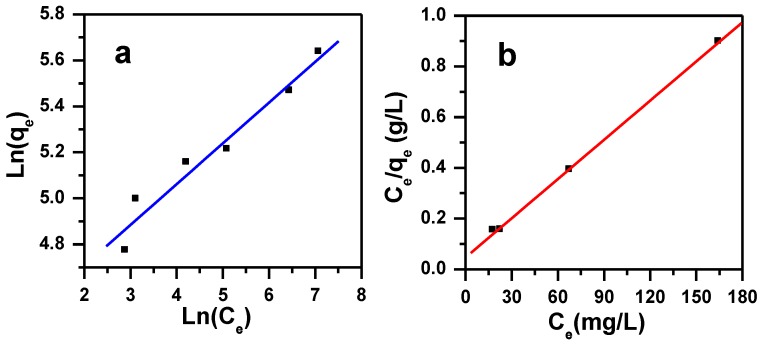
Freundlich (**a**) and Langmuir (**b**) isotherms plot for the effect of initial dye concentration on removal of MB using MNS-4.

Langmuir, Freundlich, D-R isotherm, and Temkin models were applied to fit the experimental data. The values of the regression correlation coefficients (R^2^) and the model parameters are summarized in Table 3 and presented in Figure 11. The highest value of R^2^ was obtained from Langmuir equation (0.999), and the lowest one was for the D-R model (0.822), intermediate values were obtained for Freundlich and Temkin (0.947 and 0.937). The experimental data fitted well the Langmuir model, and the removal of MB occurred on homogenous surface and formed a monolayer on the MNS-4 sample, with a maximum uptake capacity of 194 mg/g. The separation factor R_L_ was in the range of 0.0023 and 0.0076, and indicated that the removal of MB dye on modified nigella sativa is favorable.

A comparison between modified NS (MNS) and other biosorbents is presented in Table 4. 

### 3.4. Characterization and Recycling of the MNS-4 Adsorbent

#### 3.4.1. Fourier Transform Infrared Spectroscopy (FTIR)

**Figure 12:**
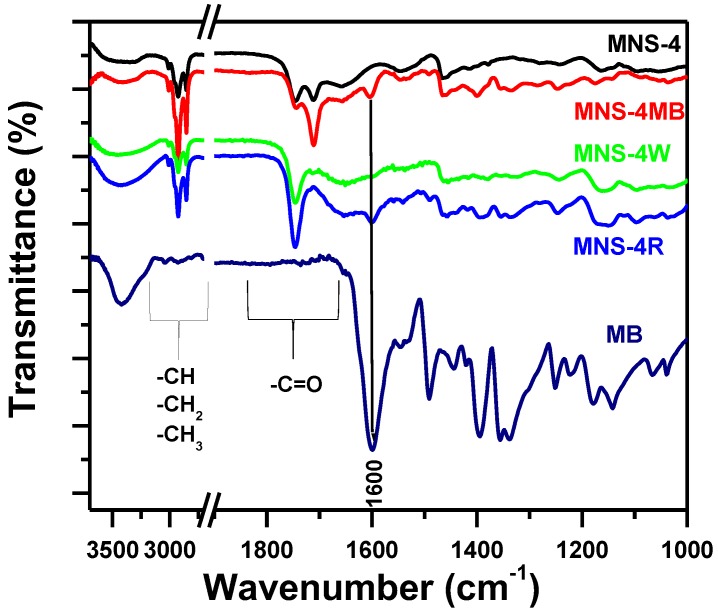
Fourier transform infrared spectroscopy (FTIR) spectra of MNS-4, MNS-4MB, MNS-4W, MNS-4R and MB.

To understand the mechanism of MB removal using MNS-4, FTIR analysis was performed on different materials before and after removal (Figure 12). The spectrum of the starting MNS-4 showed the presence of several bands located nearly at (2923–2852) cm^−1^, which correspond to C–H vibrations in methyl and methylene groups, and the bands located at (1700–1780) cm^−1^ corresponding to carbonyl groups and others functional groups [37]. While, the spectrum of MNS-4 after MB removal (MNS-4MB) exhibited an additional band at 1600 cm^−1^, related to C=C stretching of MB, indicating the presence of MB anchored to the active sites of MNS-4 adsorbent [65]. This band vanished when the solid was washed with a mixture of ethanol and water (MNS-4W), due to the removal of the MB molecules. The reused sample (MNS-4R) exhibited again the band at 1600 cm^−1^ characteristic of the MB.

#### 3.4.2. Regeneration Efficiency

**Figure 13:**
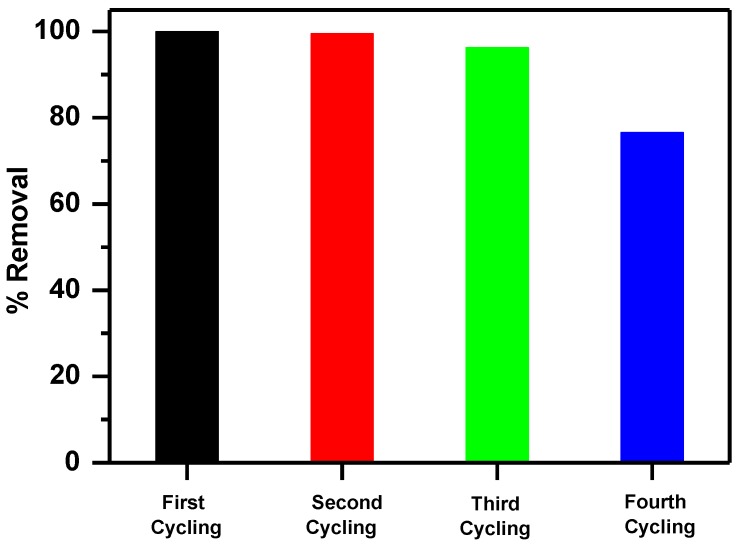
Efficiency of recycled MNS-4 for removal of MB.

The removal efficiency of MNS-4 was maintained after three cycles of regeneration cycles with an average of 99%. This value decreased to 77% for the fourth cycle (Figure 13). This decrease could indicate that some adsorption sites were blocked by the MB cations or to low desorption of the bound MB cations from MNS-4 surface [66].

#### 3.4.3. Scanning Electron Microscope (SEM)

**Figure 14:**
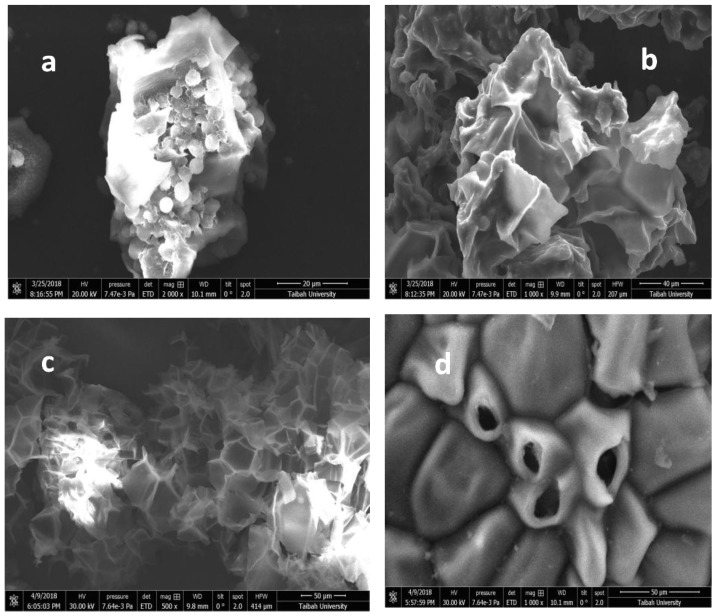
Micrographs of the scanning electron microscopy for (**a**) the parent MNS-4 and (**b**) after MB removal. (**c**) Corresponds to the regenerated material (washed with ethanol and water mixture) and (**d**) after reuse.

The changes in morphology of used materials were investigated by scanning electron microscope (Figure 14). The MNS-4 sample have heterogeneous and different spherical particles. However, after the removal of MB, the spherical shape disappeared and the surface of MNS-4 exhibited flower-like morphology, as presented in Figure 14b. The regenerated sample (washed with ethanol and water mixture) showed a significant change in morphology (Figure 14c); it exhibited alveoli morphology, leading to a honeycomb like assembly. After reuse cycle, the alveoli retracted forming hollow bags probably due to electrostatic interaction between MB and the walls of the alveoli with attraction toward the alveoli center (Figure 14d).

## 4. Conclusions

Gentle modification of nigella sativa seeds was performed, and the resulting material was tested as removal agent of MB from aqueous solutions. The removal was highly dependent on the pH, and 99% of removal efficiency was achieved for initial concentrations between 800 and 1500 ppm at pH = 11. The kinetic studies indicated that the removal of MB followed the pseudo-second order model, and the equilibrium adsorption data were better fitted to Langmuir isotherm. The maximum removal capacity was 194 mg/g as deduced from Langmuir model. Simple washing with ethanol and water mixture of the MNS-4 adsorbent was efficient to regenerate it for further reuse; the removal efficiency of 99% was maintained after three cycles of reuse and decreased to 77% after the fourth test. Modified nigella sativa seeds were proposed to be an effective and novel adsorbent, presenting an excellent performance for the removal of MB even after recycling tests.

## Figures and Tables

**Table 1:** Thermodynamic parameters for removal of MB using MNS-4.

Adsorbent	Adsorbate	∆H° (KJ·mol^−1^)	∆S° (KJ·mol^−1^·K)	∆G° (KJ·mol^−1^)		
MNS-4	MB	66	0.230	298K	313K	333K
				−2.411	−5.707	−10.761

**Table 2:** Kinetic parameters for removal of MB using MNS-4.

Dye C_i_ (ppm)	Pseudo-first Order				Pseudo-Second Order			Intra-Particle Diffusion Model		
	q_exp_ (mg/g)	q_e_ (mg/g)	K_1_ (1/min)	R_1_^2^	q_e_ (mg/g)	K_2_ (g/mg min)	R_2_^2^	I (mg/g)	K_I_ (mg/g min^0.5^)	R_3_^2^
1200	118	23	0.041	0.993	120	0.00423	1.000	99	5	0.889
1500	148	37	0.029	0.993	150	0.00214	1.000	107	4	0.937
1800	173	53	0.029	0.997	176	0.00142	1.000	116	6	0.948
2000	184	81	0.024	0.998	186	0.00077	0.998	95	9	0.989
3000	237	75	0.019	0.998	232	0.00094	0.999	151	8	0.994

**Table 3:** Isotherm parameters for removal of MB using MNS-4.

Langmuir				Freundlich			Temkin			Dubinin–Radushkevich		
q_m_ (mg/g)	K_L_ (L/mg)	R^2^	Range R_L_	q_F_ (mg^(1−1/n)^L^1/n^g^−1^)	1/n	R^2^	A_T_ (L/g)	B_T_	R^2^	q_m_ (mg/g)	R^2^	E (Kj/mol)
194	0.109	0.999	0.0023–0.0076	77	0.178	0.948	4.4E-11	0.029	0.937	85347	0.822	0.716

**Table 4:** Comparison of obtained maximum removal amount of MB (qm) with those previously reported.

Biosorbent	Q max (mg/g)	pH	Reference
Modified nigella sativa	194	11	Present work
Palm kernel fiber	95	10–11	[59]
Date stones	44	6.3	[60]
Bio-char from pyrolysis of wheat straw	12	8–9	[61]
Untreated alfa grass	200	12	[62]
Cotton stalk	111	7	[63]
Jute fiber carbon	23	5–10	[64]
